# Evaluation of pituitary function after infectious meningitis in childhood

**DOI:** 10.1186/1472-6823-14-80

**Published:** 2014-10-06

**Authors:** Claudia Giavoli, Claudia Tagliabue, Eriselda Profka, Laura Senatore, Silvia Bergamaschi, Giulia Rodari, Anna Spada, Paolo Beck-Peccoz, Susanna Esposito

**Affiliations:** Endocrinology and Diabetology Unit, Department of Clinical Sciences and Community Health, Università degli Studi di Milano, Fondazione IRCCS Ca’ Granda, Ospedale Maggiore Policlinico, Milan, Italy; Pediatric Highly Intensive Care Unit, Department of Pathophysiology and Transplantation, Università degli Studi di Milano, Fondazione IRCCS Ca’ Granda, Ospedale Maggiore Policlinico, Via Commenda 9, 20122 Milan, Italy

**Keywords:** Hypopituitarism, Meningitis, GH deficiency, Hypodrenalism, Pediatric infectious diseases

## Abstract

**Background:**

A number of studies of adults have shown that pituitary deficiencies can develop in a considerable proportion of subjects during the acute phase of meningitis or years after the infection has disappeared. The results of the very few studies of the impact of pediatric meningitis on hypothalamic-pituitary function are conflicting.

**Methods:**

In order to determine the incidence of pituitary dysfunction in children with central nervous system infection, we evaluated pituitary function and anthropometric parameters in 19 children with meningitis of different etiologies (15 males; mean age ± standard deviation [SD] at pituitary evaluation, 5.9 ± 4.0 years; mean time from the acute event ± SD, 18 ± 10 months).

**Results:**

All of the subjects had a normal stature and growth velocity for their age and gender, and none of them was obese. On the basis of Tanner’s reference charts, 17 subjects (13 boys and all four girls) were pre-pubertal; two boys were in Tanner stage 2. None of the subjects had central hypothyroidism. All of the patients had normal serum of insulin growth factor (IGF)-I and prolactin. Their sex steroid and gonadotropin levels were concordant with their age and pubertal status. Early morning urine osmolality and serum electrolyte levels showed no signs of diabetes insipidus. All of the patients had normal plasma adrenocorticotropic hormone (ACTH) levels. Peak cortisol responses to the standard dose Synacthen test (SDST) were normal in all cases.

**Conclusions:**

The results showed that hypopituitarism following infectious meningitis appears to be infrequent in childhood and children’s pituitary glands seem to be less vulnerable to damage than those of adults.

## Background

A number of studies of adults have shown that pituitary deficiencies can develop in a considerable proportion of subjects during the acute phase of viral as well as bacterial meningitis or years after the infection has disappeared [1–3]. Consequently, it has been recommended that all adults diagnosed as having meningitis should undergo pituitary function screening.

The results of the very few studies of the impact of pediatric meningitis on hypothalamic-pituitary function are conflicting. It has long been known that tuberculous meningitis causes hypothalamic-pituitary dysfunction in 20% of patients several years after their recovery [4–6], but the two studies of other forms of pediatric meningitis published found no association with the overt clinical symptoms of pituitary dysfunction [7, 8]. This has led to the conclusion that a post-meningitis clinical follow-up of growth and puberty is sufficient, and that invasive assessments are not routinely recommended [8]. However, growth failure and pubertal delay are not the only problems that can arise after hypothalamus and pituitary lesions. Secondary hypoadrenalism may often be asymptomatic and remain undiagnosed, but cause a fatal adrenal crisis during physical or psychological distress; and it is not clear whether viruses and bacteria play different roles. It is therefore necessary to investigate the real impact of meningitis on pituitary function in children in order to establish whether there are pediatric patients that need particular attention and treatment.

The aim of this study was to determine the incidence and clinical impact of pituitary dysfunction in children with a history of meningitis of different etiologies.

## Methods

The study was approved by the Ethics Committee of Fondazione IRCCS Ca’ Granda Ospedale Maggiore Policlinico in Milan, Italy, and the parents of all of the participants gave their written informed consent.

After taking a detailed history in order to exclude any pre-meningitis symptoms suggesting endocrinal problems, we consecutively enrolled 19 patients with a history of infectious meningitis hospitalized at the Pediatric Highly Intensive Care Unit, Fondazione IRCCS Ca’ Granda Ospedale Maggiore Policlinico, Milan, Italy, from January 1, 2013, to December 31, 2013: 15 males; mean age at pituitary evaluation 5.9 ± 4.0 years; mean time from the acute event 18 ± 10 months (Table 1). The diagnosis of meningitis was based on clinical symptoms and cerebrospinal fluid examinations and cultures for bacteria as well as polymerase chain reaction (PCR) for viruses. The etiology was viral in eight patients confirmed by PCR (enterovirus n = 5; varicella zoster virus n = 3) and bacterial in 11 confirmed by culture (*Streptococcus pneumoniae* n = 4, *Neisseria meningitidis* n = 2, *Streptococcus agalactiae* n = 2, *Staphylococcus aureus* n = 1, *Haemophilus influenzae* type b n = 1, *Mycobacterium tuberculosis* n = 1). All the patients had a Glasgow Coma Scale ≥14.

**Table 1:** **Growth parameters, and clinical and demographic characteristics of children with a previous diagnosis of acute meningitis**

Patient No.	Gender/age (years)	Height (SDS)	BMI (SDS)	Tanner stage	Etiology of meningitis	Time from acute event (months)
**1**	M/10.1	-1.1	-0.63	2	Enterovirus	3
**2**	M/2.8	1.6	0.69	1	Enterovirus	33
**3**	M/6.5	1.0	-0.14	1	Enterovirus	15
**4**	F/6.3	2.0	0.44	1	Enterovirus	15
**5**	F/3.9	0.9	0.01	1	Enterovirus	16
**6**	M/3.5	0.2	0.41	1	Varicella zoster virus	8
**7**	M/6.9	-0.7	-0.45	1	Varicella zoster virus	12
**8**	M/3.3	1.3	0.98	1	Varicella zoster virus	23
**9**	M/13.9	0.4	0.65	3	*Streptococcus pneumoniae*	32
**10**	F/3.6	0.6	0.41	1	*Streptococcus pneumoniae*	29
**11**	M/6.3	-0.4	0.00	1	*Streptococcus pneumoniae*	11
**12**	M/4.9	1.0	-0.55	1	*Streptococcus pneumoniae*	37
**13**	F/8.9	0.8	-1.42	1	*Neisseria meningitidis*	13
**14**	M/12.9	1.9	2.28	2	*Neisseria meningitidis*	12
**15**	F/1.5	-0.4	-0.51	3	*Streptococcus agalactiae*	15
**16**	M/1.1	1.2	-1.14	1	*Streptococcus agalactiae*	10
**17**	M/1.1	-1.1	-1.58	1	MRSA	6
**18**	M/3.2	2.0	-1.58	1	*Haemophilus influenzae* type b	32
**19**	M/12	0.3	-0.63	1	*Mycobacterium tuberculosis*	18

M = male; F = female; SDS = standard deviation scores; BMI = body mass index, MRSA: methicillin-resistant *Staphylococcus aureus*.

Patients’ height and weight were used to calculate their body mass index (BMI), and the results were compared with the WHO gender- and age-related curves. Height velocity was determined using a previous measurement made at least six months before the beginning of the study and preserved in the patients’ medical records; parental height was also measured in order to calculate target height. Standard deviation scores (SDS) were used for the anthropometric measurements, and the Tanner and Whitehouse reference charts were used for pubertal staging [9].

After 3 to 37 months (median, 15 months) from the acute event, baseline blood samples were taken after an overnight fast and used to measure the levels of adrenocorticotropic hormone (ACTH), insulin growth factor (IGF)-I, prolactin (PRL), sodium and potassium, and thyroid and gonadal function. An early morning urine sample was tested for osmolality. The hypothalamic-pituitary-adrenal (HPA) axis was evaluated by means of a standard dose Synacthen test (SDST: ACTH 125 μg in children aged <2 years; 250 μg in children aged ≥2 years) administered as an intravenous bolus at baseline, with serum cortisol levels being measured after 0, 30 and 60 minutes; central hypoadrenalism was excluded by the presence of a cortisol peak >20 μg/dL (550 nmol/L) [10]. Serum thyroid stimulating hormone (TSH) and free thyroid hormone levels (FT4 and FT3) were compared with the normal age-adjusted reference ranges supplied by the hospital’s central laboratory; central hypothyroidism was ruled out by the presence of normal serum FT4 and FT3 serum levels [11]. The SDS of IGF-I were calculated using age- and gender-related reference ranges. The levels of gonadotropins and sex steroids (estradiol or testosterone) were interpreted on the basis of the subjects’ pubertal stage.

Serum cortisol levels were measured using an electrochemiluminescence immunoassay (ECLIA, Roche Cobas Cortisol, Roche Diagnostics, Mannheim, Germany) with an inter-assay coefficient of variation of 1.4-1.6% and an intra-assay coefficient of variation of 1.0-1.4%. Serum IGF-I levels were measured using a chemiluminescent immunometric assay (Immulite 2000 IGF-I; Siemens Medical Solutions Diagnostics, Los Angeles, CA), with intra- and interassay coefficients of variation of respectively 2.9% and 7.4%. Serum FT4, FT3 and TSH concentrations were measured using electrochemiluminiscent immunoassays (Roche Diagnostics). All of the other parameters were measured using standard methods.

After using Levene’s test to check the equality of variances, the normally distributed data were expressed as mean values ± standard deviation (SD).

## Results

All of the subjects had a normal stature (mean height SDS ± SD 0.6 ± 0.9) and growth velocity for their age and gender, and none of them was obese (mean BMI SDS ± SD 0 ± 1). On the basis of Tanner’s reference charts [9], 17 subjects (13 boys and all four girls) were pre-pubertal (stage B1 or a testicular volume of <4 mL); two boys were in Tanner stage 2 (testicular volume 8 mL) (Table 1).

Table 2 summarises the main laboratory parameters. None of the subjects had central hypothyroidism as shown by the normal serum levels of FT4 and FT3 (mean FT4 ± SD 12.9 ± 2.1; mean FT3 ± SD 4.5 ± 0.5). All of the patients had normal serum of IGF-I (mean IGF-I SDS ± SD -0.9 ± 0.5) and prolactin, and their sex steroid and gonadotropin levels were concordant with their age and pubertal status. Early morning urine osmolality and serum electrolyte levels (mean sodium ± SD 139 ± 0.2 mEq/L; mean potassium ± SD 4.6 ± 0.3 mEq/L) showed no signs of diabetes insipidus.

**Figure 1:**
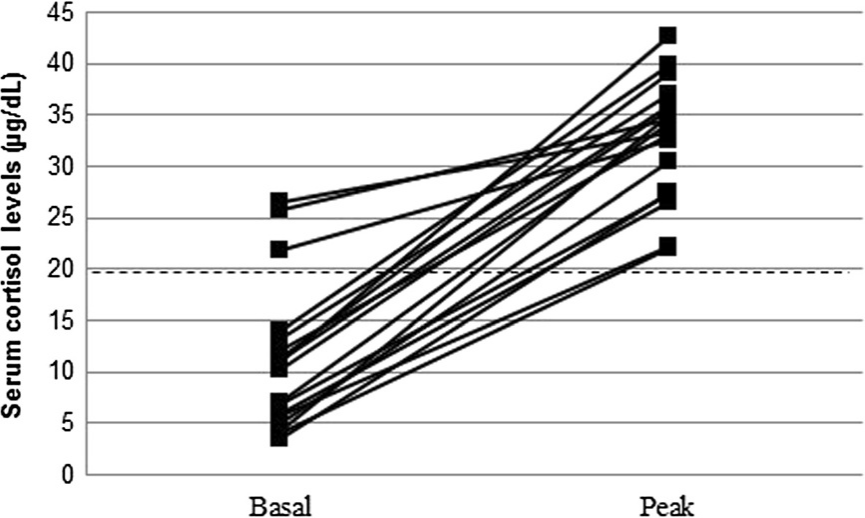
**Serum cortisol levels during SDST (standard dose Synachten test) in each patient.** Cut-off for normal peak: 20 μg/dL (dashed line).

All of the patients had normal plasma ACTH levels (mean ACTH ± SD 22.1 ± 9.6 pg/mL). Mean basal cortisol levels ± SD were 10.7 ± 7.3 (normal values 6.2-19.4 μg/dL), and slightly low in four subjects, but peak cortisol responses to the SDST were normal in all cases, including those with low basal levels (mean cortisol peak ± SD 32.2 ± 5.9; normal values >20 μg/dL). Figure 1 shows the cortisol response to SDST in each patient.

**Table 2:** **Main laboratory parameters**

Patient No.	IGF-I (SDS)	TSH mcIU/ml (n.v. 0.6-7.6)	FT4pg/mL (n.v. 8–17)	FT3 pg/mL (n.v. 2.5-5.3)	PRL ng/mL (n.v. 1.7-16)	Potassium mEq/L (n.v. 3.5-5.1)	Sodium mEq/L (n.v. 135–145)
**1**	-0.35	1.81	17.5	4.4	7.1	3.8	135
**2**	-1.18	5.19	16.6	5.4	10.6	3.9	139
**3**	-1.49	2.66	10.4	3.8	15.1	3.9	140
**4**	-0.15	1.8	13.2	4	2.6	4.2	139
**5**	-1.18	2.05	12.1	4.1	7.4	5	139
**6**	-1.09	2.18	11.9	4.8	5.0	3.7	131
**7**	-0.15	1.7	14	5.2	13.7	4.7	143
**8**	-0.47	2.15	13.6	4.1	13.6	4.8	140
**9**	-0.98	2.44	10.8	3.9	7.4	4.7	142
**10**	-1.50	2.66	10.4	3.8	15.1	4.3	140
**11**	-1.25	2.72	13	4.5	7.4	4.2	139
**12**	-1.46	1.29	13.5	4.4	8.7	4.2	140
**13**	-1.17	4.56	13.2	4.3	5	4.4	143
**14**	-1.29	2.17	9.8	4.3	1.9	4.8	141
**15**	-1.51	1.58	13.3	4.6	14.8	4.3	139
**16**	-1.46	2	11.6	4.4	13.4	4.8	138
**17**	-1.51	2.88	15.9	5.5	9.1	4.4	138
**18**	-0.57	2.27	11.4	5.2	5.6	4.9	137
**19**	-0.11	1.69	12.8	4.4	2.9	4.5	138

SDS = standard deviation scores; n.v. = normal values; IGF-I = insulin-like growth factor I; TSH = thyroid- stimulating hormone; FT4 = free T4; FT3 = free T3; PRL = prolactin.

## Discussion

Despite the relatively small number of subjects, the findings of this study seems to support the hypothesis that pediatric meningitis does not impair childhood pituitary function, regardless of its viral or bacterial etiology. This is different from the findings in adultswith viral as well as bacterial meningitis, which frequently indicate post-meningitis ACTH and growth hormone (GH) deficiency [1–3]. The difference seems to reflect an age-related difference in susceptibility to pituitary damage as previous studies of adults [12] and children [13] have shown that, similarly to findings observed in meningitis, pituitary dysfunction after traumatic brain injury (which is observed in 33% of adults after three months and in 22% after 12 months) is also significantly less likely in children.

Unlike Levy-Shraga et al. [7], who only studied basal pituitary function, we excluded the presence of post-meningitis central hypoadrenalism by dynamically evaluated the HPA axis using the safe and simple SDST, which has previously been used by Karadag-Oncel et al. [8]. Basal cortisol levels are not always useful in diagnosing central hypoadrenalism mainly because they do not reveal any reduction in pituitary ACTH reserve. Furthermore, and unlike Karadag-Oncel et al. [8], we used a glucagons test to assess adrenal function because it is more reliable than the gold standard insulin tolerance test [14]. The SDST may not always be effective in establishing or excluding the presence of central hypoadrenalism in children [8]. The post-SDST cortisol peak reached by each patient was greatly above the established cut-off value definitely excluding central hypoadrenalism, and none of the subjects complained of any symptoms of adrenal insufficiency.

Regarding the hypothalamic-pituitary-gonadal axis, 17 of our 19 patients were pre-pubertal and so possible hypogonadism could not be revealed by means of a clinical or hormonal evaluation. However, the two boys in Tanner stage 2 had normal testosterone and gonadotropins levels.

In relation to the other pituitary axis, none of the patients showed hyper- or hypo-prolactinemia, and central hypothyroidism was ruled out by the presence of normal free thyroid hormone levels.

Post-meningitis GH deficiency has been described and confirmed by provocative tests in adults [1–3]. We did not evaluate GH secretion dynamically because of all of the children had normal height, growth velocity, and IGF-I SDS. However, GH deficiency cannot be definitely excluded without a longer follow-up and, if necessary, dynamic testing.

A limitation of the study is represented by the heterogeneous aetiology of the cases as well as by the fact that none of them had severe neurologic involvement. It could be possible that in complicated cases as tuberculous meningitis or neonatal meningitis and in presence of severe neurologic deterioration the results could be different.

## Conclusions

The findings of this study show that hypopituitarism following infectious meningitis is significantly less frequent in children than in adults. Like those observed after traumatic brain injury, the data suggest that the pituitary of children may be less susceptible to damage. Moreover, there did not seem to be any difference between the effects of meningitides of viral or bacterial etiology. However, the relatively small number of enrolled children means that our findings need further confirmation, particularly the role of the different etiological agents. Furthermore, the finding of hypopituitarism in young adults with a history of childhood meningitis suggests that, although the systematic endocrine evaluation and specific examinations of children diagnosed as having meningitis may not be necessary, their long-term clinical evaluation with registration of height and weight would make it possible to identify the few cases in which pituitary alterations may occur.

## Abbreviations

ACTH: Adrenocorticotropic hormone
BMI: Body mass index
ECLIA: Electrochemiluminescence immunoassay
FT: Free thyroid hormone levels
GH: Growth hormone
HPA axis: Hypothalamic-pituitary-adrenal axis
IGF-I: Insulin growth factor-I
PCR: Polymerase chain reaction
PRL: Prolactin
SD: Standard deviation
SDS: Standard deviation scores
SDST: Standard dose Synacthen test
TSH: Serum thyroid stimulating hormone.
